# Rational use of inhaled corticosteroids for the treatment of COPD

**DOI:** 10.1038/s41533-023-00347-6

**Published:** 2023-07-24

**Authors:** Jennifer K. Quint, Amnon Ariel, Peter J. Barnes

**Affiliations:** 1 National Heart and Lung Institute, Imperial College London, London, UK; 2 Lung Unit, Emek Medical Center, Afula, Israel

**Keywords:** Chronic obstructive pulmonary disease, Outcomes research, Respiratory signs and symptoms

## Abstract

Inhaled corticosteroids (ICS) are the mainstay of treatment for asthma, but their role in chronic obstructive pulmonary disease (COPD) is debated. Recent randomised controlled trials (RCTs) conducted in patients with COPD and frequent or severe exacerbations demonstrated a significant reduction (~25%) in exacerbations with ICS in combination with dual bronchodilator therapy (triple therapy). However, the suggestion of a mortality benefit associated with ICS in these trials has since been rejected by the European Medicines Agency and US Food and Drug Administration. Observational evidence from routine clinical practice demonstrates that dual bronchodilation is associated with better clinical outcomes than triple therapy in a broad population of patients with COPD and infrequent exacerbations. This reinforces guideline recommendations that ICS-containing maintenance therapy should be reserved for patients with frequent or severe exacerbations and high blood eosinophils (~10% of the COPD population), or those with concomitant asthma. However, data from routine clinical practice indicate ICS overuse, with up to 50–80% of patients prescribed ICS. Prescription of ICS in patients not fulfilling guideline criteria puts patients at unnecessary risk of pneumonia and other long-term adverse events and also has cost implications, without any clear benefit in disease control. In this article, we review the benefits and risks of ICS use in COPD, drawing on evidence from RCTs and observational studies conducted in primary care. We also provide a practical guide to prescribing ICS, based on the latest global treatment guidelines, to help primary care providers identify patients for whom the benefits of ICS outweigh the risks.

## Introduction

Inhaled corticosteroids (ICS) have long been the mainstay of asthma treatment, improving symptom control and reducing the risk of serious exacerbations^1^. In contrast, there is much debate about the role of ICS relative to long-acting inhaled bronchodilators, i.e. long-acting muscarinic antagonists (LAMAs) and long-acting β_2_-agonists (LABAs), which in combination represent the mainstay of treatment for chronic obstructive pulmonary disease (COPD)^2–4^.

Although ICS are effective in some patients with COPD, they are less effective in many others. The major clinical benefit of ICS in COPD is a ~25% reduction in exacerbations in frequent or severe exacerbators, with no significant benefits in terms of lung function or mortality^5,6^. ICS-containing regimens are associated with a higher risk of pneumonia vs. single or dual long-acting bronchodilator therapy, as well as a higher risk of oropharyngeal candidiasis, mycobacterial infections and upper respiratory tract infections^7,8^. There is also evidence to suggest that long-term ICS use is associated with systemic adverse effects such as reduced bone mineral density (osteopenia), osteoporosis, fractures, diabetes, skin thinning and bruising, as well as ocular adverse effects such as cataract formation and glaucoma^8–11^.

**Fig. 1:**
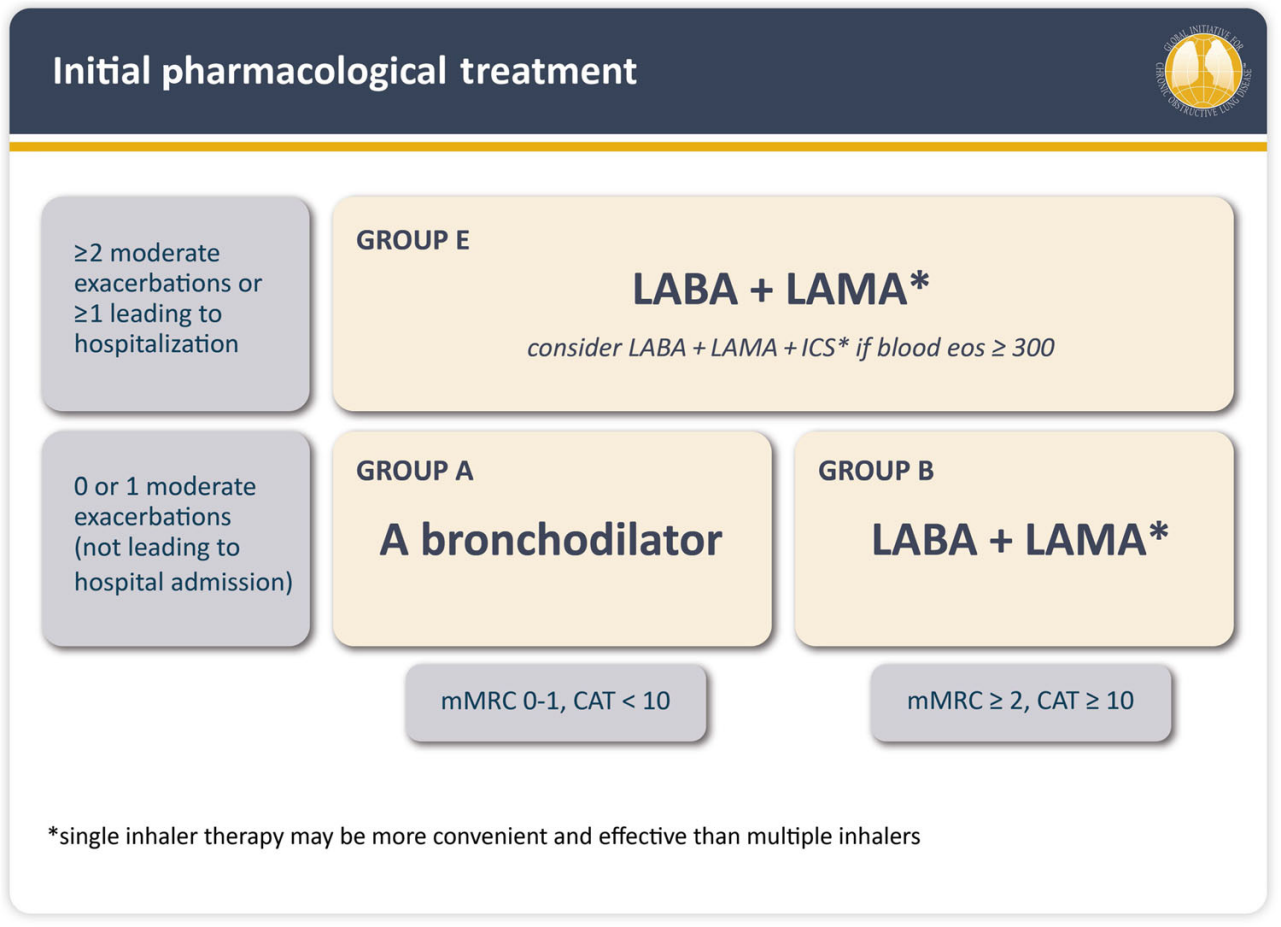
GOLD 2023. Initial pharmacological treatment. *Single inhaler therapy may be more convenient and effective than multiple inhalers. Groups C and D from GOLD 2011–2022 have been replaced by Group E in GOLD 2023^4^. CAT COPD Assessment Test, Eos eosinophils, GOLD Global Initiative for Chronic Obstructive Lung Disease, ICS inhaled corticosteroids, LABA long-acting β_2_-agonist, LAMA long-acting muscarinic antagonist, mMRC modified Medical Research Council. © 2022, 2023, Global Initiative for Chronic Obstructive Lung Disease, available from www.goldcopd.org, published in Deer Park, IL, USA.

Since 2007, the Global Initiative for Chronic Obstructive Lung Disease (GOLD) strategy report has shifted its pharmacotherapy focus from a spirometry-based approach relying on the assessment of forced expiratory volume in 1 s (FEV_1_; GOLD stages 1–4, GOLD 2007) to an exacerbation and symptom-based algorithm (GOLD grades A–D, GOLD 2011 to 2022; GOLD grades A/B/E, GOLD 2023) (Fig. 1)^4,12,13^. These changes occurred concurrently with an evolving understanding of the relatively narrow, phenotype-specific benefits of ICS use in COPD^14,15^. Hence, the 2023 GOLD strategy report includes a practical recommendation for initial treatment with ICS (as LAMA/LABA/ICS; triple therapy) in patients with frequent or severe exacerbations (≥2 moderate exacerbations per year, or ≥1 exacerbation requiring hospitalisation) and a blood eosinophil count ≥300 cells/μl^4^. In patients with blood eosinophils ≥100 cells/μl who continue to have exacerbations despite LAMA/LABA therapy, treatment can be escalated to triple therapy, after careful consideration of the expected benefits vs. risks. While the use of LABA/ICS is no longer encouraged in COPD^4^, if patients with COPD have concomitant asthma, the use of ICS is mandatory^4^.

Although the proportion of frequent exacerbators in the COPD population is generally less than 30%, and the proportion of eosinophilic exacerbators even lower (~10%)^16–23^, observational studies show that ICS-containing pharmacotherapy is prescribed in around 50–80% of patients with COPD^20,22,24–27^. Possible factors contributing to the overuse of ICS in COPD are suggested in Table 1. Over-prescription of ICS is illustrated by findings from an analysis of 1528 patients with COPD in Europe, in which only 10.6% of patients were found to have a blood eosinophil count of ≥300 cells/μl and a history of ≥2 moderate/≥1 severe exacerbations in the previous 12 months^22^. Despite GOLD recommendations indicating that ICS use should be limited to this subset of patients^4^, as many as 41.5% of GOLD B patients and 68.0% of GOLD D patients were receiving ICS in this study^22^. ICS overuse is also highlighted by observational data from the COPDGene (*n* = 1553) and ECLIPSE (*n* = 1895) studies, in which ICS were used by as many as 48% and 71% of patients with eosinophils <300 cells/μl, respectively^20^. In addition, many studies have shown that more patients receive ICS-containing therapies at initiation of first maintenance therapy than would be expected based on their exacerbation history^20,22,24–31^. The consequences of this are serious, as overtreatment with ICS is associated with a significant health-economic burden associated with the increased risk of adverse events such as pneumonia and higher treatment costs^32^.

**Table 1:** Possible factors contributing to ICS overuse in patients with COPD.

Reason
• Delayed/late introduction of LAMA to market, e.g., vs. LABA/ICS^118^ • Overstated importance of ICS-responsive exacerbations in COPD^21,28^ • Perceived similarity of asthma and COPD, leading to assumption that as ICS are effective in asthma, they will also be effective in COPD^25,119^ • Co-existence of asthma and COPD (either real or due to diagnostic confusion) leading to prescription of ICS^31,32,118,120^ • Exaggerated perception of LABA/ICS benefits in COPD, including hope that the ICS component can reduce the impact of symptoms^32,120^ • Lack of confidence in bronchodilators to prevent exacerbations, despite available evidence to the contrary^31^ • Difficulty for physicians in recognising the benefits of long-acting bronchodilators, which may be subtle but meaningful in the long term^120^ • Poor familiarity of prescribing physicians with GOLD recommendations and treatment guidelines for appropriate ICS use^31,120^ • Strong influence of physicians’ personal prescribing preferences^31^ • Downplaying the impact of ICS adverse events, e.g., based on the reduced side-effect profile associated with low–moderate doses used in asthma^32^
• Randomised controlled trials of triple therapy claiming major benefits in terms of exacerbation and survival vs. dual therapy^54,56^

*COPD* chronic obstructive pulmonary disease, *GOLD* Global Initiative for Chronic Obstructive Lung Disease, *ICS* inhaled corticosteroids, *LABA* long-acting β_2_-agonist, *LAMA* long-acting muscarinic antagonist.

In this article, we briefly summarise evidence for the effectiveness and safety of ICS in COPD, gleaned from both randomised controlled trials (RCTs) and observational studies. We comment on patient characteristics guiding recommended use and the factors contributing to the ongoing overuse of ICS in COPD. We also include strategies for safe ICS withdrawal. Lastly, we provide a practical guide to appropriate ICS prescribing, to help primary care providers identify those patients for whom the benefits of ICS are likely to outweigh the risks. In doing so, we hope to enable and encourage evidence-based use of ICS in COPD.

## ICS use in asthma versus COPD

ICS are absorbed systemically from the peripheral lung and exert their immunosuppressant effects locally in the upper and lower airways, as well as potentially inducing systemic immunosuppressant effects. In most patients with asthma, low doses of ICS are highly effective in reducing chronic eosinophilic inflammation of the airways^33^. However, the lung inflammation characteristic of COPD is predominantly neutrophilic^34^. This difference in inflammatory endotype accounts for corticosteroid resistance in the majority of patients with COPD, with no effect on inflammation, disease progression or mortality, and only a small improvement in spirometry/reduction in exacerbations^33,35,36^. Only a minority of patients with COPD have an eosinophilic phenotype^37–41^ and can therefore be considered corticosteroid sensitive, as reflected in COPD treatment guidelines.

Although the term “asthma-COPD overlap” is no longer in widespread use, patients with COPD can have concomitant asthma. Prevalence estimates vary, and some evidence suggests that asthma is over-diagnosed in patients with COPD^42^. However, in cases where COPD and asthma are confirmed to coexist, pharmacotherapy should predominantly follow asthma guidelines (i.e. prescription of ICS)^4,42^.

Key points

- ICS are very effective in treating asthma, even at low doses, due to their effects on eosinophilic inflammation.
- In most patients with COPD, airway inflammation is not eosinophilic, and even high doses of ICS have poor efficacy.
- ICS use should be limited to patients with eosinophilic COPD and those with concomitant asthma.

## Randomised controlled trials of ICS in COPD

### ICS monotherapy vs. placebo

Although ICS use has been studied more extensively in asthma, many RCTs have evaluated the efficacy and safety of ICS in COPD (Table 2)^7^. A Cochrane review of treatment with ICS alone (55 primary studies, including >16,000 patients) found that ICS did not modify the long-term decline of FEV_1_ or mortality in patients with COPD^43^. This review noted a relatively small but statistically significant reduction in the mean exacerbation rate with ICS. However, the rate of pneumonia increased by >50% in the ICS group. Furthermore, in the TORCH trial, a trend toward higher mortality was observed for patients treated with ICS (fluticasone propionate) alone^44^. Consequently, the GOLD 2011 report recommended against using ICS monotherapy in COPD^45^.

**Table 2:** Strengths and limitations of selected RCTs of ICS in patients with COPD.

Pharmacotherapy	Key trials/reference (year)	Key findings	Main critique	Reference to GOLD COPD documents
ICS alone	Yang IA, et al. Cochrane Database Syst Rev (2012)^43^	RCTs of ICS vs. placebo >6 months: • Modest decrease in exacerbations • No decrease in FEV_1_ decline or mortality • Increased pneumonia risk	Exacerbation benefit overestimated due to ICS withdrawal effect^47^	GOLD Report 2011 “ICS monotherapy not recommended in COPD as it is less effective than LABA/ICS (Evidence A)”^45^
LABA/ICS	TORCH (2007)^44^ • 3-year survival study • Moderate–severe COPD (FEV_1_ < 60%), SAL/FP500 vs. its mono-components and placebo	All-cause mortality (complete survival follow-up): • HR LABA/ICS vs. placebo 0.825 (95% CI 0.681, 1.002), *p* = 0.052) • Primary endpoint not achieved • Incomplete (discontinuation) follow-up for all non-primary (non-survival) endpoints Pneumonia events: • LABA/ICS and ICS groups (18–20%) • LABA and placebo groups (12–13%)	• Placebo is not “usual care” • 2-week run-in with ICS and LABA withdrawal (28% dropout) • ICS withdrawal (45–50%) • LABA withdrawal (35%) • Factorial (2*2 analysis) not published by study authors but otherwise calculated to show survival benefit attributable to LABA, not ICS, component^47^	GOLD REPORT 2023—Efficacy of ICS alone^4^ “In the TORCH trial, a trend toward higher mortality was observed for patients treated with fluticasone propionate alone compared to those receiving placebo or salmeterol plus fluticasone propionate combination”
Adding LAMA	Canadian OPTIMAL Study (2007)^49^ • 52-week, 3-arm exacerbation RCT • Moderate–severe COPD (FEV_1_ < 65%); ≥1 exacerbation/year; past asthma excluded - LAMA (Tio, *n* = 156) - LAMA/LABA (Tio/SAL; *n* = 148) - LAMA + LABA/ICS (Tio + SAL/FP250; *n* = 145)	• Proportion of patients who experienced moderate–severe exacerbations: - LAMA (62.8%) - LAMA + LABA (64.8%) - LAMA + LABA/ICS (60%) - Rate ratios not significantly different • Hospitalisations lower in LAMA + LABA/ICS vs LAMA group: - HR 0.67 (95% CI 0.45, 0.99) • 40% premature discontinuation in non-ICS arms	• First trial of triple therapy in COPD, non-pharma sponsored • Complete (intent-to-treat) follow-up • High withdrawal rate in non-ICS arms related to ICS withdrawal on randomisation (75% pre-study ICS use) • No exacerbation benefit in ICS-naïve subjects (per-study non-users)^47^	Study not cited
LAMA/LABA vs. LABA/ICS	FLAME (2016)^50^ • 52-week, 2-arm exacerbation RCT - Moderate–severe COPD (FEV_1_ 25–60%); >1 exacerbation/year; past asthma excluded. - LAMA/LABA (GLY/IND; *n* = 1680) - LABA/ICS (SAL/FP250; *n* = 1682)	• 4-week LAMA (Tio) run-in associated with 32% dropout rate • Annual rate of all COPD exacerbations: - 11% lower in LAMA/LABA group than LABA/ICS group (RR 0.89; 95% CI 0.83, 0.96; *p* = 0.003) • Incidence of pneumonia: - 3.2% in LAMA/LABA group and 4.8% in LABA/ICS group	• Study design tending to exclude ICS responders • Run-in bias (4-week Tio run-in; all ICS and LABA discontinued) • Past asthma excluded • Subjects with blood eosinophil count >600 cells/µl excluded • The reported HR for time to first exacerbation may represent a magnification of the real effect^51^	No referral to this study regarding ICS use in COPD

*CI* confidence interval, *COPD* chronic obstructive pulmonary disease, *FEV*_*1*_ forced expiratory volume in 1 s, *FP* fluticasone propionate, *GOLD* Global Initiative for Chronic Obstructive Lung Disease, *HR* hazard ratio, *ICS* inhaled corticosteroids, *LABA* long-acting β_2_-agonist, *LAMA* long-acting muscarinic antagonist, *RCT* randomised controlled trial, *RR* relative risk, *SAL* salmeterol, *Tio* tiotropium.

### LABA/ICS vs. placebo

From 2000 onwards, many landmark trials have evaluated the efficacy of ICS in combination and comparison with long-acting bronchodilators^7^. In 2007, TORCH demonstrated a significant reduction in exacerbations and improvement in health status and lung function with LABA/ICS vs. placebo in patients with COPD—a finding that was replicated for exacerbations and lung function in the SUMMIT trial in 2016^44,46^. However, both of these large-scale trials failed to achieve their primary objective: a statistically significant, ICS-related reduction in mortality. Moreover, a post hoc factorial analysis of the TORCH trial showed a survival benefit associated with the LABA, but not the ICS, component (Table 2)^47^. Like many COPD studies, both trials were confounded by ICS withdrawal prior to randomisation and incomplete post-discontinuation follow-up for all their secondary end points, including exacerbations^47,48^.

### LABA/ICS vs. LAMA/LABA/ICS

The Canadian OPTIMAL trial, an independent, non-industry-sponsored trial, notable for its complete exacerbation follow-up, investigated the efficacy of adding LABA/ICS to LAMA in patients with moderate-to-severe COPD, and demonstrated a reduction in exacerbations that did not reach statistical significance^49^. A post hoc analysis of OPTIMAL demonstrated that the apparent decrease in exacerbations was limited to pre-study ICS users and thus largely attributable to ICS discontinuation on randomisation^47^.

### LABA/ICS vs. LAMA/LABA

In 2016, the FLAME study highlighted the benefits of LAMA/LABA, finding a reduced annual rate of moderate or severe exacerbations and lower incidence of pneumonia vs. LABA/ICS^50^. However, the superiority of LAMA/LABA over LABA/ICS demonstrated in FLAME was probably exaggerated by its inclusion criteria and 4-week run-in with LAMA and withdrawal of ICS, which is likely to have preserved more LAMA responders relative to ICS responders (Table 2)^51^.

### LAMA/LABA/ICS vs. LAMA/LABA

Following the shift towards more LAMA/LABA use instead of LABA/ICS between 2013 and 2018, the last 5 years have seen a resurgence in ICS use after the approval of triple-therapy inhalers based on the results of pivotal studies such as TRIBUTE, IMPACT, KRONOS and ETHOS^5,6,52,53^. IMPACT and ETHOS have reported the benefits of using LAMA/LABA/ICS vs. LAMA/LABA in patients with COPD and a high exacerbation risk in terms of reducing exacerbations and mortality, albeit with a higher incidence of pneumonia with triple therapy^5,6^. However, various publications have commented extensively on methodological issues with the design and analysis of these studies in terms of the populations studied, the confounding effect of ICS withdrawal prior to randomisation, and the inclusion of patients with a history of asthma (Table 3)^54–58^. While all three trials are confounded by ICS withdrawal on randomisation, this effect is further magnified in IMPACT and ETHOS (vs. TRIBUTE) by the selective inclusion of frequent exacerbators with non-severe airflow limitation (FEV_1_ > 50%, GOLD 2D), thereby selecting an unusual asthma-like, “ICS-sensitive” study population compared with the general COPD population^59^. Additionally, in both KRONOS and ETHOS, the trial design included a 1–4-week screening period in which withdrawal of all long-acting bronchodilators induced a very large pre-randomisation dropout rate (38% and 46%, respectively), thus favouring the selective inclusion of “ICS-sensitive” subjects^6,53^. Thus, for IMPACT and ETHOS, the reported survival benefit in the ICS arms is largely attributable to the transient effect of ICS withdrawal on randomisation in a selected “ICS-sensitive” cohort^56,60^.

**Table 3:** Strengths and limitations of RCTs comparing LAMA/LABA with triple therapy.

Study	TRIBUTE 2018^52^	IMPACT 2018^5^	ETHOS 2020^6^	KRONOS^53^
Population	≥40-year-old patients with COPD, CAT ≥ 10, non-current (past) asthma—allowed^a^			
	• FEV_1_ < 50% predicted and ≥1 exacerbation/year • 65% pre-study ICS users • 0% pre-study triple therapy (excluded)	• FEV_1_ 50–80% predicted and ≥2 (or ≥1 severe) exacerbation/year, OR—FEV_1_ < 50% predicted and ≥1 exacerbation/year - 71% pre-study ICS users - 38% pre-study triple therapy (allowed)	• FEV_1_ 50–65% predicted and ≥2 (or ≥1 severe) exacerbation/year, OR—FEV_1_ 25–50% predicted and ≥1 exacerbation/year • 80% pre-study ICS users • 39% pre-study triple therapy (allowed) • ~60% of enrolled patients had a blood eosinophil count of ≥150 cells/mm^3^	• FEV_1_ 25–80%; prior exacerbations not required • 71% pre-study ICS users • 27% pre-study LAMA/LABA/ICS • 27% ≥1 or more moderate/severe exacerbations in previous year
Study arms	• LAMA/LABA/ICS (BDP/FORM/GLY; *n* = 764) • LAMA/LABA (IND/GLY; *n* = 768)	• LAMA/LABA/ICS (FF/UMEC/VI; *n* = 4151) • LABA/ICS (FF/VI; *n* = 4134) • LAMA/LABA (UMEC/VI; *n* = 2070)	• LAMA/LABA/high-dose ICS (BUD320/GLY/FORM; *n* = 2157) • LAMA/LABA/low-dose ICS (BUD160/GLY/FORM; *n* = 2137) • LABA/high-dose ICS (BUD320/FORM; *n* = 2151) • LAMA/LABA (GLY/FORM; *n* = 2143)	• LAMA/LABA/ICS (BDP/FORM/GLY; *n* = 640) • LAMA/LABA (FORM/GLY *n* = 627) • LABA/ICS (BUD/FORM pMDI *n* = 316) • LABA/ICS open-label (BUD/FORM DPI *n* = 319)
Design	52-week double-blind RCT	52-week double-blind RCT	52-week double-blind RCT	24-week double-blind RCT
	Run-in: 2 weeks LAMA/LABA (IND/GLY)	No run-in	During variable 1–4-week screening period: • Per-study ICS continued • LAMA/LABA replaced by ipratropium 2 puffs QID and albuterol rescue with 46% drop-out—no details provided	During variable 1–4-week screening period: • Per-study ICS continued • LAMA/LABA replaced by ipratropium 2 puffs QID and albuterol rescue with 38% drop-out—no details provided
Findings: Exacerbations & pneumonia	• Decreased adjusted moderate–severe exacerbations • BDP/FORM/GLY vs. IND/GLY - RR 0.848 (95% CI 0.723, 0.995; *p* = 0.043) • RR of moderate and severe exacerbations analysed separately not significantly different • Pneumonia events: Similar pneumonia rates (4%) in ICS/non-ICS groups (BDP/FORM/GLY; IND/GLY)	• Decreased adjusted moderate–severe exacerbations • FF/UMEC/VI vs. FF/VI - RR 0.85 (95% CI 0.80, 0.90; *p* < 0.001) • FF/UMEC/VI vs. UMEC/VI - RR 0.75 (95% CI 0.70, 0.81; *p* < 0.001) • Pneumonia events: - FF/UMEC/VI; FF/VI (7–8%) vs. UMEC/VI (5%) - HR 1.53 (95% CI 1.22, 1.92; *p* < 0.001). (FF pneumonia rates higher than BUD160–320; see ETHOS)	• Decreased adjusted moderate–severe exacerbations • BUD160 and 320/GLY/FORM vs. BUD320/FORM - BUD160/GLY/FORM: RR 0.86 (95% CI 0.79, 0.95; *p* = 0.002) - BUD320/GLY/FORM: RR 0.87 (95% CI 0.79, 0.95; *p* = 0.003) • BUD160 and 320/GLY/FORM vs. GLY/FORM - BUD160/GLY/FORM: RR 0.75 (95% CI 0.69, 0.83; *p* < 0.001) - BUD320/GLY/FORM: RR 0.76 (95% CI 0.69, 0.83; *p* < 0.001) • Pneumonia events: - BUD/GLY/FORM (3.5–4.2%); BUD/FORM (4.5%) vs. GLY/FORM (2.3%) - BUD160/GLY/FORM vs. BUD320/GLY/FORM achieved similar results with less pneumonia risk (3.5% vs. 4.2%)	Decreased moderate or severe exacerbations (secondary endpoint) • BGF MDI vs. GFF MDI: rate ratio 0.48 (95% CI 0.37, 0.64; *p* < 0.0001) • BGF MDI vs. BFF MDI: rate ratio 0.82 (0.58, 1.17; *p* = 0.2792) • BGF MDI vs. BUD/FORM DPI: rate ratio 0.83 (0.59, 1.18; *p* = 0.3120)
Findings: Mortality	Study not powered for mortality	Mortality^121^: • FF/UMEC/VI (2.36%) vs. UMEC/VI (3.19%) - HR for death 0.72 (95% CI 0.53, 0.99; *p* = 0.042) • FF/UMEC/VI (2.36%) vs. FF/VI (2.64%) - HR for death 0.89 (95% CI 0.67, 1.16; *p* = 0.387)	Mortality^122^ • BUD320/GLY/FORM vs. BUD320/FORM - HR 0.72 (95% CI 0.44, 1.16; *p* = 0.1721) • BUD320/GLY/FORM vs. GLY/FORM - HR 0.51 (95% CI 0.33, 0.80; *p* = 0.0035)	Not powered for mortality
Critique	• Customary study population (exacerbators with FEV_1_ < 50%); allowing non-current asthma • 2-week LAMA/LABA run-in • Prior triple therapy: 0% • Prior ICS therapy: 65% • ICS withdrawal (mixed intervention)	• Unusual study population: - allowing non-current asthma, - inclusion of frequent exacerbators without severe airflow limitation (asthma like, FEV_1_ > 50% GOLD 2D sub-cohort) • Withdrawal of prior ICS (71%) and triple therapy (38%); mixed intervention • Both exacerbation^56^ and mortality^60^ benefit essentially confined to the first 90 days of the study—representing ICS withdrawal in an “ICS-sensitive” sub-cohort • Mortality indication rejected by regulatory agencies^116,117^	• Unusual study population: - allowing non-current asthma, - inclusion of frequent exacerbators without severe airflow limitation (asthma like, FEV_1_ > 50% GOLD 2D sub-cohort) • Excluding patients with very severe airflow limitation (FEV_1_ < 25%) • Unusual 1–4-week pre-randomisation screening with long-acting bronchodilator withdrawal and 46% dropout, suggestive of significant run-in bias^51^ • Withdrawal of prior ICS (80%) and triple therapy (39%)—mixed intervention • Mortality benefit^60^ essentially confined to the first 90 days of the study—representing ICS withdrawal in an “ICS-sensitive” sub-cohort	• Allowed non-current asthma • 71% of patients on ICS therapy prior to study • Run-in withdrawal of long-acting bronchodilators associated with 38% pre-randomisation drop-out

^a^Population criteria same across all four studies.

*BDP* beclometasone dipropionate, *BGF* budesonide/glycopyrrolate/formoterol fumarate, *BUD* budesonide, *CAT* COPD Assessment Test, *CI* confidence interval, *COPD* chronic obstructive pulmonary disease, *DPI* dry powder inhaler, *FEV*_*1*_ forced expiratory volume in 1 s, *FF* fluticasone furoate, *FORM* formoterol fumarate, *GFF* glycopyrrolate/formoterol fumarate, *GLY* glycopyrronium, *GOLD* Global Initiative for Chronic Obstructive Lung Disease, *HR* hazard ratio, *ICS* inhaled corticosteroids, *IND* indacaterol, *LABA* long-acting β_2_-agonist, *LAMA* long-acting muscarinic antagonist, *MDI* metered dose inhaler, *OR* odds ratio, *pMDI* pressurised metered dose inhaler, *QID* four times/day, *RCT* randomised controlled trial, *RR* relative risk, *UMEC* umeclidinium, *VI* vilanterol.

The confounding effect of ICS withdrawal is a recurrent limitation in the RCTs of triple therapy. In an optimally designed trial, patients on triple therapy at screening would be excluded outright, and other patients would be randomly allocated to specific treatment arms based on their current therapy^54,55^. However, in the absence of such trials, observational studies from routine clinical practice can provide valuable evidence by investigating the long-term effect of ICS in larger cohorts of “new users”, avoiding the effect of medication switching/withdrawal on randomisation and better representing the overall population of COPD patients.

Key points

- The major clinical benefit of ICS in COPD is a ~25% reduction in exacerbations, observed in RCTs of patients with frequent or severe exacerbations.
- Though some RCTs suggest that adding ICS to LAMA/LABA or LABA therapy increases survival in patients with COPD, methodological flaws in these trials have led regulatory authorities to dismiss claims of survival benefit associated with ICS.
- The study design and populations involved in the pivotal studies of ICS in COPD were very specific and did not represent many patients with COPD, focusing a priori on a small subset of patients who stand to benefit the most from ICS treatment.

## Observational and other studies of ICS in COPD

Patients with COPD in primary care may differ significantly from patients enrolled in large-scale RCTs in terms of characteristics such as gender, lung function, quality of life and exacerbations^61^. Observational studies may provide a more balanced picture as they generally represent a broader population of patients with COPD compared with the narrow subgroups of patients studied in clinical trials such as IMPACT and ETHOS. For example, in the DACCORD observational study, which evaluated LAMA/LABA/ICS (*n* = 1046) vs. LAMA/LABA (*n* = 1046) in patients initiating or changing their COPD maintenance therapy, LAMA/LABA was associated with a lower proportion of patients experiencing an exacerbation compared with LAMA/LABA/ICS (15.5% vs. 26.6%; *p* < 0.001). A greater improvement from baseline in COPD Assessment Test total score (mean ± standard deviation −2.9 ± 5.8 vs. −1.4 ± 5.5; *p* < 0.001) and a greater proportion of patients having a clinically relevant improvement (61.8% vs. 47.2%; *p* < 0.001) were also observed^21^.

**Fig. 2:**
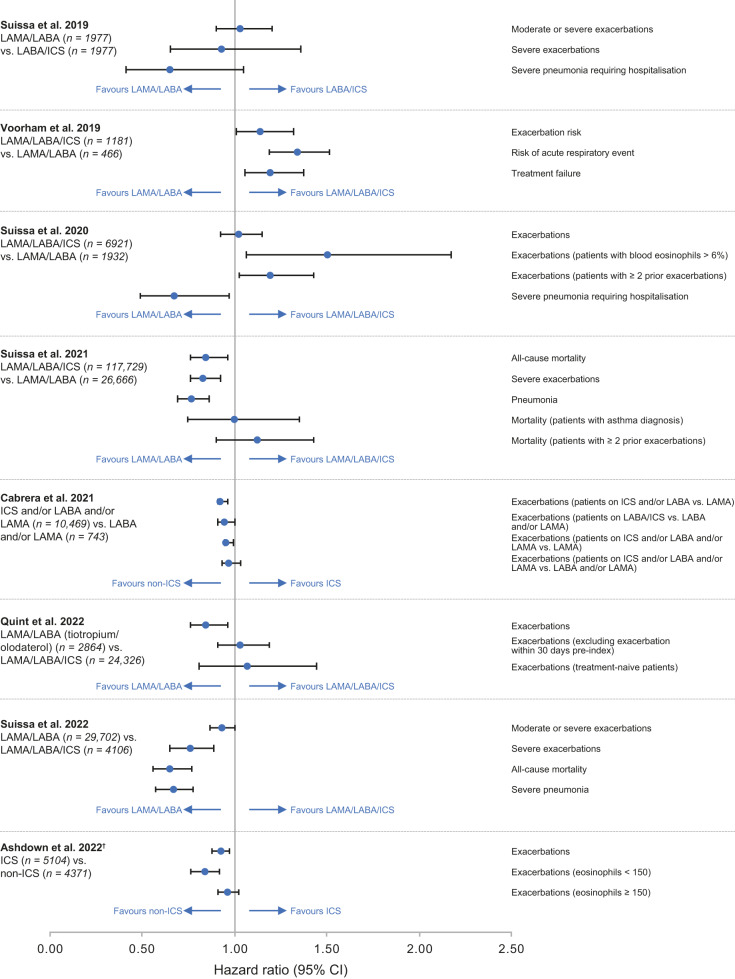
Real-world studies comparing LAMA/LABA with triple therapy or LABA/ICS in patients with COPD*. *Data from Voorham et al.^62^, Suissa et al.^64^^,^^65^^,^^68^, Cabrera et al.^66^ and Ashdown et al.^86^ transformed for consistent presentation (favours non-ICS-containing therapy on left; favours ICS-containing therapy on right). ^†^HRs are for time to first event after treatment initiation. Suissa et al.^63^, patients aged >55 years initiating LAMA/LABA or LABA/ICS; Voorham et al.^62^, patients aged >40 years with a history of smoking and no maintenance/LAMA therapy and ≥2 exacerbations in the previous year; Suissa et al.^64^, patients aged >55 years initiating LAMA/LABA/ICS or LAMA/LABA; Suissa et al.^65^, patients aged >50 years initiating LAMA/LABA/ICS or LAMA/LABA; Cabrera et al.^66^, patients initiating ICS vs. non-ICS-containing treatments; Quint et al.^67^, patients aged ≥40 years with ≥1 prescription of LAMA/LABA/ICS or LAMA/LABA; Suissa et al.^68^, patients aged ≥40 years initiating treatment with single-inhaler LAMA/LABA/ICS, or LAMA/LABA. CI confidence interval, COPD chronic obstructive pulmonary disease, HR hazard ratio, ICS inhaled corticosteroids, LABA long-acting β_2_-agonist, LAMA long-acting muscarinic antagonist.

Findings from other observational studies comparing LAMA/LABA with triple therapy or LABA/ICS in COPD are summarised in Fig. 2. Of these, only one study replicated the findings of IMPACT/ETHOS (see Voorham et al.^62^). However, similar to IMPACT and ETHOS, these patients had a history of ≥2 exacerbations in the preceding year^62^, which is not representative of the general COPD population. Conversely, studies conducted in broader, more representative populations, i.e. less frequent exacerbators and less severe COPD^21,63–68^, have not replicated the findings of IMPACT and ETHOS, showing a similar or lower risk of exacerbations, mortality and pneumonia in patients receiving non-ICS treatment (Fig. 2). In a post hoc pooled analysis including Phase III and IV trials with duration ≥12 months in the tiotropium/olodaterol clinical programme (in which ICS continuation was permitted), no difference in mortality was found between LAMA/LABA and LAMA/LABA/ICS over 52 weeks^69^. The population of patients with mild-to-very-severe COPD was predominantly of lower exacerbation risk than either IMPACT or ETHOS, excluded patients with a history of asthma and did not include ICS withdrawal^69^.

Observational evidence also suggests that elevated blood eosinophil counts may predict COPD exacerbation risk in ex-smokers, but not current smokers^70^. However, there is a possibility of selection bias in such trials as it is not known what triggered blood testing in the first place. Reduced sensitivity to ICS in current vs. ex-smokers has also been shown in analyses of the SUMMIT, IMPACT and FLAME studies^71–73^, and in a pooled analysis of data from ILLUMINATE, LANTERN and FLAME^74^.

Key points

- Data from observational studies, which are generally conducted in broader populations and are more representative of clinical practice, provide no evidence that the benefits of ICS observed in RCTs are generalisable to the COPD population as a whole.
- Current smokers with COPD are likely to have a reduced sensitivity to ICS compared with ex-smokers.

## Identifying ICS responders in COPD

### Exacerbation frequency/phenotype

Global treatment guidelines for COPD state that patients with frequent or severe exacerbations (≥2 moderate exacerbations per year, or ≥1 exacerbation requiring hospitalisation) and a blood eosinophil count ≥300 cells/μl are more likely to benefit from the addition of ICS^4^. However, when identifying frequent exacerbators, it is not just the frequency of exacerbations that is important, but also the type of exacerbation. Not all exacerbations are the same and they have different underlying triggers, which unlike eosinophilic inflammation, are not steroid responsive. For example, 50–70% of exacerbations are attributed to respiratory infections, 10% to environmental pollution and up to 30% are of unknown aetiology^75^. Notably, hospital admissions for COPD exacerbations nearly halved during the COVID-19 pandemic, likely due to a reduction in respiratory viral infections triggering exacerbations^76^. Comorbidities such as heart failure or gastroesophageal reflux may also be drivers of exacerbations, as well as mental health conditions such as anxiety and depression^75,77,78^.

Before considering use of ICS in frequent exacerbators, it is important to consider the phenotype and cause of the exacerbations in order to tailor treatment, rather than taking a “one-size-fits-all” approach to treatment^73,79,80^.

### Eosinophil threshold

Analysis of several RCTs has shown an association between eosinophil count and ICS responsiveness in terms of preventing future exacerbations when used in combination with long-acting bronchodilators in patients with COPD^5,52,81–84^. Some post hoc analyses of RCTs have suggested that the benefit of ICS begins at a blood eosinophil threshold of 100 cells/μl^72,81^. However, the ETHOS results bring into question this threshold as no treatment benefit of triple therapy vs. non-ICS dual therapy on the annual rate of moderate or severe exacerbations was observed in a subgroup analysis of patients with eosinophils <150 cells/μl (at either dose of budesonide)^6^. In the IMPACT study, the authors state that the benefits of triple therapy on the annual rate of moderate/severe exacerbations were seen regardless of eosinophil level, though they acknowledge a greater reduction in exacerbation rate in the ≥150 cells/μl subgroup^5^. In a post hoc analysis of data from IMPACT, triple therapy was associated with a lower exacerbation rate vs. LAMA/LABA in patients with eosinophils ≥100 cells/μl, but only in those with a history of frequent moderate or severe exacerbations. In patients with a history of a single moderate exacerbation, a lower exacerbation rate in the triple therapy arm was only observed at higher eosinophil levels (≥200 cells/μl)^85^.

Since findings on eosinophil thresholds can be affected by selection bias for certain patient populations (such as frequent exacerbators) in RCTs, as described earlier in this article, the threshold derived from observational studies may be more reliable. Several observational studies have shown that the optimal blood eosinophil count threshold for ICS response in terms of the ability to reduce exacerbations is considerably higher (300–450 cells/μl)^86–88^ than findings from RCTs (100–150 cells/μl)^5,6,72,81^. However, other analyses have not replicated these findings. In a systematic review of 11 RCTs and 5 observational studies, an overall association between blood eosinophil count and the effect of ICS in reducing exacerbation risk was found. However, this association was not observed in four of the five observational studies, suggesting that the predictive effect of eosinophils may not apply to the real-world COPD population^89^.

An observational study comparing initial COPD treatment with LABA/ICS or LAMA suggests that while the exacerbation benefit of initiating LABA/ICS is demonstrated only in patients with high blood eosinophil counts (>300 cells/μl), the increased pneumonia risk with LABA/ICS is observed at all eosinophil concentrations^87^. Based on this, the authors conclude that initial treatment with a LAMA should be preferred in patients with blood eosinophil concentrations ≤300 cells/μl due to its superior risk/benefit profile^87^. A blood eosinophil threshold of ≥300 cells/μl forms the basis of the guidance for initial treatment of COPD in the GOLD 2022 strategy report, aiming to identify exacerbating COPD patients that are likely to derive the greatest benefit from ICS^4^.

Key points

- It is important to assess the number and type of exacerbations before prescribing ICS to patients with COPD (ICS reduce eosinophilic exacerbations but not infection-based exacerbations).
- In patients with a history of frequent or severe exacerbations, global treatment guidelines recommend starting with dual bronchodilator therapy (LAMA/LABA).
- Addition of ICS to LAMA/LABA is recommended for frequent/severe exacerbators with blood eosinophil levels ≥300 cells/µl (or ≥100 cells/µl if exacerbations are not well controlled by LAMA/LABA).

## Increased risk of adverse events and cost implications associated with ICS therapy

### Pneumonia

The higher risk of pneumonia in COPD patients treated with ICS is well documented and is acknowledged in the GOLD 2023 report^4^. In a systematic review of 19 RCTs by Miravitlles et al., exposure to ICS for ≥1 year increased the risk of pneumonia by 41% (risk ratio 1.41, 95% confidence interval 1.23–1.61)^8^. The risk varied according to the type of ICS used, with fluticasone propionate or furoate having the highest risk (10 studies: *n* = 45,870)^8^. Conversely, exposure to budesonide (six studies: *n* = 13,479) was not associated with an increased risk of pneumonia, although a high degree of heterogeneity was observed due to one large study that reported an increased risk^8^. These findings are in contrast to a European Medicines Agency Pharmacovigilance Risk Assessment Committee review of the known risk of pneumonia in patients with COPD receiving ICS, which reported no conclusive evidence of differences in the risk of pneumonia between different products^90^. However, in support of the Miravitlles systematic review, a large observational analysis of 39,362 new users of triple therapy (either fluticasone- or budesonide-based) showed a lower incidence of severe pneumonia in patients on budesonide-containing regimens compared with fluticasone^91^.

As shown by one US database study of 135,445 patients with COPD, the use of ICS in newly diagnosed patients was associated with a dose-related increase in the risk of pneumonia^30^. In an observational study of patients recruited from routine clinical practice in the UK (the Salford Lung Study), mortality after admission with pneumonia was higher than after admission with an exacerbation, suggesting that pneumonia may have a greater impact on survival than exacerbations^92^. Real-world data such as these are important in the interpretation of fatal pneumonia risk associated with ICS use in COPD, as data from randomised controlled trials are often confounded by the exclusion of patients at highest risk of pneumonia, e.g. those with low lung function, very low BMI or significant comorbidities^30^. However, it should also be acknowledged that definitions of pneumonia and acute exacerbations of COPD often overlap within electronic health records. As such, they may not always be as rigorously differentiated from each other as they should be, compared with clinical trials, in which an X-ray diagnosis of pneumonia is often a requirement.

Variations in the risk of pneumonia and other adverse events associated with ICS use in patients with COPD may possibly be explained by their effects on the composition of the lung microbiome^93–98^. Several studies have reported changes in airway microbiome composition following ICS treatment, including reductions in α-diversity, increases in sputum bacterial load/modification of sputum microbial composition and increased airway load of potentially pathogenic bacteria, e.g., increased risk of acquiring the respiratory pathogen *Pseudomonas aeruginosa*^93,94,97,98^. However, further studies are needed to clarify the effects of ICS on the lung microbiome. Most recently, ICS withdrawal in the INCOGNITO study was associated with potentially beneficial changes in microbiome composition and altered the exacerbation endotype, with a reduction in bacterial-associated exacerbations^99,100^. The increased risk of respiratory infections with ICS treatment may be linked with their immunosuppressive effects, including reductions in T-cell, macrophage and neutrophil function in the lung^101,102^.

### Other adverse events

In addition to the increased risk of pneumonia, both cohort and nested case–control studies show an association between ICS use and the risk of tuberculosis and mycobacterial disease^8^. A strong association has also been reported between ICS use and local disorders such as oral candidiasis and dysphonia, although the association with diabetes and bone fractures is less clear and appears significant only at high doses of ICS^8,9^. Some studies have found a significantly increased risk of cataracts associated with cumulative ICS exposure but results from other studies did not find a significant association^8^. Of note, elderly patients with COPD have an increased risk of osteoporosis and cataracts^11^ and non-elderly patients with COPD have an increased risk of osteoporosis^101,103,104^. ICS also carries an increased risk of developing type 2 diabetes in patients with COPD, particularly at high doses^105^.

### Cost implications

Triple therapy containing ICS may not be the most cost-effective approach, especially as first-line treatment and in patients with less severe COPD. Compared with dual therapy, triple therapy is associated with significant increases in hospitalisation rates and cost of care^106^. Inappropriate prescription of ICS is associated with poorer physical health status as well as higher costs of COPD management^107^. Several studies have shown that reducing inappropriate ICS use and increasing use of LAMA/LABA resulted in better outcomes, including a reduction in exacerbations and pneumonia cases, accompanied by lower total COPD costs^108,109^. As first-line treatment, LAMA/LABA is also associated with significantly lower pharmacy costs attributable to COPD or pneumonia vs. triple therapy and is more cost-effective than triple therapy in patients in GOLD groups A/B vs. GOLD groups C/D^67^.

Key points

**Fig. 3:**
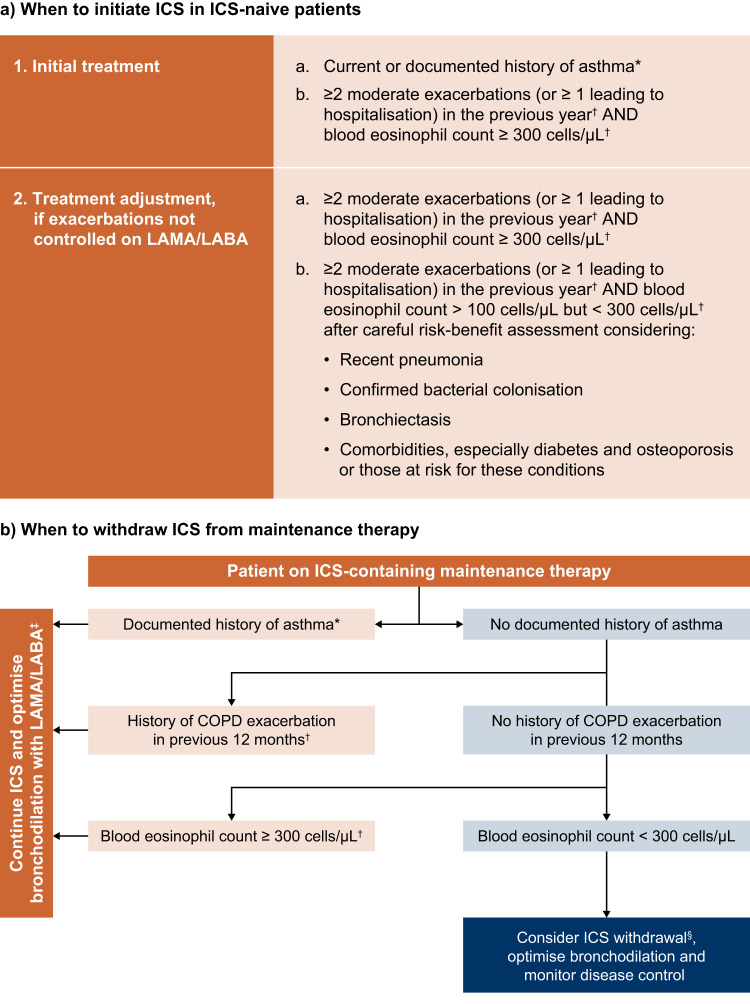
Practical guide to prescribing ICS for the treatment of COPD. Adapted from the International Primary Care Respiratory Group (IPCRG) desktop helper for the appropriate use and withdrawal of ICS, 2020. Available at link. *This may include asthmatic features/features suggesting steroid responsiveness, including any previous, secure diagnosis of asthma or atopy, a higher blood eosinophil count, substantial variation in FEV_1_ over time (at least 400 ml) or substantial diurnal variation in peak expiratory flow (at least 20%). ^†^Or since previous assessment if less than 12 months. ^‡^For patients with exacerbations despite triple therapy (LAMA/LABA/ICS), consider add-on therapy with roflumilast or macrolides. ^§^If blood eosinophil count is 150–300 cells/μl, reduce ICS dose/switch to an ICS with a better safety profile. If blood eosinophil count is <150 cells/µl, and there is no/questionable asthma history or exacerbation in the previous 12 months, consider withdrawal as risks of ICS are likely to outweigh any benefit. COPD chronic obstructive pulmonary disease, FEV_1_ forced expiratory volume in 1 s, ICS inhaled corticosteroids, LABA long-acting β_2_-agonist, LAMA long-acting muscarinic antagonist.

- ICS-containing therapies increase the risk of many unwanted side effects, in particular, pneumonia, in patients with COPD.
- Because of this, it is important to consider the benefits vs. the risks of treatment when prescribing ICS.
- Unnecessary use of ICS has cost implications for primary care providers.
- We have included a practical guide to the appropriate prescription of ICS, to help identify patients for whom the benefits of ICS are likely to outweigh the risks (Fig. 3).

## ICS withdrawal

**Fig. 4:**
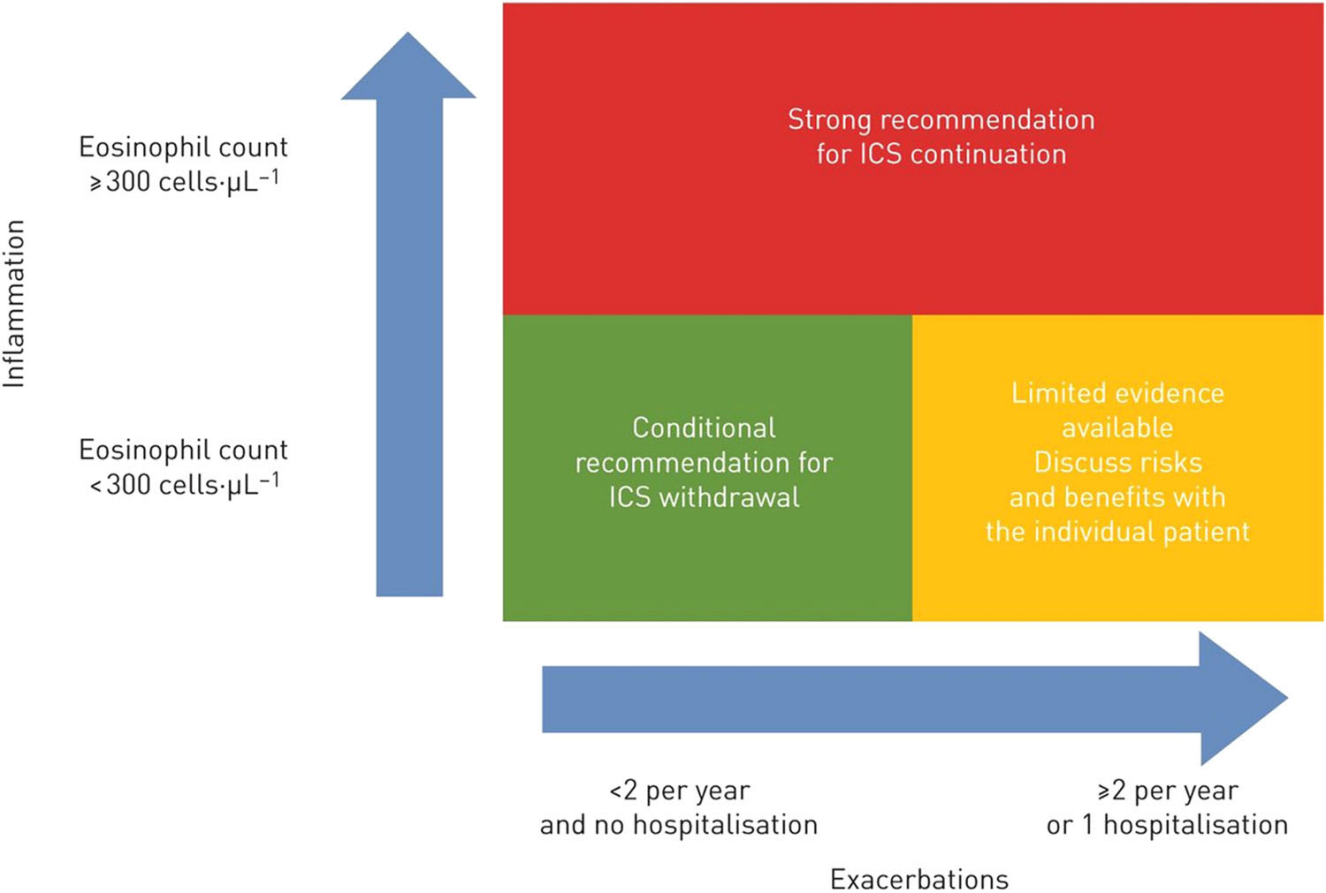
Summary of the European Respiratory Society guidelines on withdrawal of ICS in COPD^110^. COPD chronic obstructive pulmonary disease, ICS inhaled corticosteroids. Reproduced with permission of the © ERS 2022. *European Respiratory Journal* 55 (6) 2000351; 10.1183/13993003.00351-2020 Published 4 June 2020.

In an effort to reverse the trend of over-prescribing, ICS withdrawal should be considered. For example, the European Respiratory Society (ERS) guidelines recommend ICS withdrawal in patients with COPD who do not have a history of frequent exacerbations (Fig. 4)^110^. Similarly, the American Thoracic Society conditionally recommends ICS withdrawal in patients with COPD receiving triple therapy if the patient has had no exacerbations in the past year^111^. The ERS guidelines strongly recommend that ICS should not be withdrawn in patients with blood eosinophil counts ≥300 cells/μl^110^. For patients with an eosinophil count <300 cells/μl, withdrawal is conditionally recommended, taking into account patient view and benefits vs. risks. The rationale for withdrawal is clearer at an eosinophil count <150 cells/μl, if there is no history of exacerbations and the patient is receiving no objective benefit from ICS^110^.

Studies have shown that ICS withdrawal does not have a detrimental effect in patients at a low risk of exacerbations when sufficient bronchodilation is in place^27,112^. For example, in an observational study of >85,000 patients with COPD who initiated LAMA/LABA/ICS therapy, there was a lower incidence of severe COPD exacerbations requiring hospitalisation in those who discontinued ICS treatment compared with those who continued; however, this was not the case in patients with a history of frequent exacerbations or asthma^112^. Other observational studies have reported similar findings. In a study of 48,157 patients with elevated blood eosinophil counts, there was no increased risk of moderate and/or severe COPD exacerbations or all-cause mortality among subjects that withdrew from ICS^113^. In a study of 11,093 patients with COPD that withdrew from ICS for ≥6 months, 69% of patients had no recorded exacerbation event and 89% had no hospitalisation for COPD during the withdrawal^114^. In a non-interventional study of ~1200 patients, in which physicians identified patients who could be “stepped down” from triple therapy to LAMA/LABA, no overall decline in COPD was observed and outcomes improved in some cases^115^. Although coding of ICS withdrawal and clinical outcomes may differ between observational studies, these findings suggest that ICS can be safely withdrawn in many patients who are currently prescribed triple therapy, leaving them on bronchodilator therapy.

Key points

- Withdrawal of ICS should be considered in patients who do not fulfil guideline criteria for ICS use (Fig. 3).
- If a patient’s eosinophils are low (<150 cells/µl), they have not exacerbated in the previous year, and there is no or questionable asthma history, then consider withdrawal, as risks of ICS are likely to outweigh any benefit.
- When sufficient bronchodilation is in place, ICS withdrawal does not have a detrimental effect in patients at a low risk of exacerbations.

## Conclusions: recommendations for ICS in the COPD treatment paradigm

ICS overuse continues despite the narrow, specific recommendations for ICS use in global COPD guidelines, and the introduction of single-inhaler triple therapy is associated with a resurgence of this concerning trend.

Recent RCTs of single-inhaler triple therapy have demonstrated significant exacerbation and survival benefits, but this is largely attributed to ICS withdrawal in frequent exacerbators, representing a minority of COPD patients. As a result, healthcare regulatory bodies have rejected claims of survival benefit associated with triple therapy^116,117^. Observational studies conducted in broader, more representative COPD populations have demonstrated that patients treated with LAMA/LABA have a similar or more often lower risk of exacerbations, mortality and pneumonia compared with patients treated with ICS^64–66^. In patients not fulfilling guideline criteria for ICS use, prescription of ICS puts them at unnecessary risk of pneumonia and other long-term adverse events, without any clear benefit in disease control. Inappropriate use of ICS also has cost implications for the management of COPD. ICS should therefore be reserved for the few, not the many, i.e. those with an eosinophilic, frequent/severe exacerbator phenotype. In patients not fulfilling guideline criteria, ICS should be withdrawn in line with global treatment guidelines.

### Reporting summary

Further information on research design is available in the Nature Research Reporting Summary linked to this article.

## Supplementary information

Reporting summary

## Data Availability

All data included in the review are sourced from published information in the public domain.
